# Natural Products Self-Assembled Nanozyme for Cascade Detection of Glucose and Bacterial Viability in Food

**DOI:** 10.3390/foods10112596

**Published:** 2021-10-27

**Authors:** Qiuping Zhang, Xinze Wang, Yi Kang, Hao Sun, Yanmin Liang, Jie Liu, Zehui Su, Jie Dan, Linpin Luo, Tianli Yue, Jianlong Wang, Wentao Zhang

**Affiliations:** College of Food Science and Engineering, Northwest A&F University, Xianyang 712100, China; Zhangqp777@163.com (Q.Z.); wxz1628022031@163.com (X.W.); kky9628@163.com (Y.K.); 15591898565@163.com (H.S.); liangyymm@163.com (Y.L.); liujie@nwafu.edu.cn (J.L.); suzehui0809@163.com (Z.S.); danjie921@163.com (J.D.); linpinluo@nwafu.edu.cn (L.L.); yuetl@nwsuaf.edu.cn (T.Y.); wanglong79@nwsuaf.edu.cn (J.W.)

**Keywords:** nanozyme, cascade reaction, glucose concentration, bacterial viability

## Abstract

Sugar content and bacterial contamination levels are important indicators for the health and safety of food, respectively. Therefore, it is important to construct a method that can detect both glucose and bacterial activity. Herein, natural compounds (gallic acid and glucose oxidase) were assembled into nanozyme (GOx@GA-Fe (ii)) for mild cascade detection. The nanozyme catalyzes glucose to produce hydrogen peroxide, which is then converted to ·OH and oxidized colorless TMB from blue oxidized TMB. Under the optimal conditions, the method has a good linear relationship in the glucose concentration range of 1–500 μM (R^2^ = 0.993) with minimum detection concentration of 0.43 μM. Based on the glucose consumption of bacteria metabolism, the cascade reaction was then applied to detect the viability of 5 common bacteria. As such, a cascade method based on a natural self-assembled nanozyme was fabricated to monitor the quality of food.

## 1. Introduction

Soft drinks are nonalcoholic drinks that contain up to 45% glucose and are a favorite among consumers. Soft drinks contain a lot of sweeteners, including glucose, sucrose, fructose, and artificial additives. However, compared with other sugars, glucose is abundant and common in daily life, which might be a continuous modifiable risk factor for eye, kidney, and peripheral nerve disease [1]. In addition, glucose is the most common oligosaccharide that provides energy for metabolism and aids in the digestion and mediation of macromolecular nutrients. Although foods high in glucose can make people feel good, they also increase the risk of diseases, such as diabetes. Therefore, soft drinks have been linked to diabetes; long-term consumption of soft drinks can lead to diabetes and nonalcoholic fatty liver disease [2]. If we do not take urgent action, 578 million people are predicted to suffer from diabetes by 2030, and 700 million by 2045 [3,4]. There is no cure for diabetes. Preventive treatment is the most effective way to adjust patients’ habits and reduce risks via real-time monitoring of blood sugar [5,6]. Therefore, the detection of sugar content in food is of great significance for the control of human health.

Colorimetry is a commonly used detection method that can be observed by the naked eye. Because this method is convenient and does not require precise instruments, it has great potential for routine detection methods [7,8,9]. Biological colorimetry sensors include gold nanoparticles, which have been widely used to monitor various foods [10,11,12]. A tandem enzyme is an enzyme with two or more catalytic functions that can catalyze cascade reactions [13,14,15]. With the development of nanotechnology, nanozymes (also known as enzyme-like nanomaterials) have been widely evaluated as substitutes for natural enzymes in analytical chemistry, catalysis, and cancer treatment due to their advantages: simple preparation process, low cost, stability, and high catalytic activity [16,17]. Many glucose-based cascading enzymes have been reported, for example, GOx@CuBDC [18] and GOx@ZIF-8 (NIPD) [19]. When glucose is present, the enzymes catalyze glucose to produce hydroxyl free radicals. In addition, TMB is oxidized, and the oxidized TMB gives the reaction a blue color [20,21]. However, cascading enzymes require complex synthesis methods and expensive raw materials, which limit their practical application for detection [22,23,24].

Besides, there is a risk of microbial contamination in the production, processing, storage, transportation, and sale of food, including soft drinks. According to the World Health Organization, foodborne diseases are among the most important public health problems worldwide, especially in developing countries, due to poor medical conditions and unsanitary environments [25,26]. Therefore, a fast, efficient method for detecting bacteria is also necessary.

**Scheme 1:**
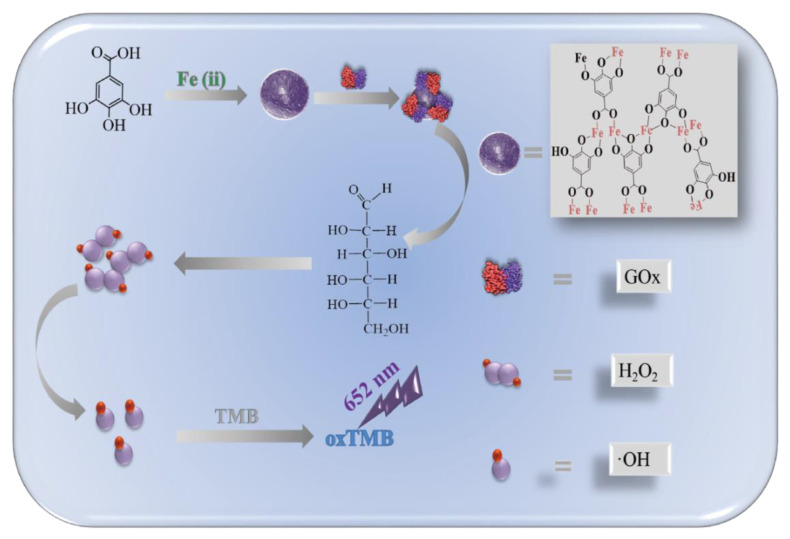
The synthesis process of GOx@GA-Fe (ii) and its principle in glucose detection.

In this study, we developed a method for the cascade detection of glucose and viable bacteria in food by generating a stable structure from gallic acid and ferrous ions, which were then connected to glucose oxidase to form the self-assembled nanozyme GOx@GA-Fe (ii). The large surface area of metal-organic framework provides metal ions for Fenton reaction simultaneously. The structure is not only suitable for loading macromolecules, but also has good biocompatibility and biodegradation ability. The nanozyme synthesis method is simple and fast and requires mild synthesis conditions. It can catalyze glucose to obtain its concentration by TMB colorimetry. Scheme 1 is a schematic of the synthesis of the nanomaterial and outlines its catalysis of glucose via the following three-step cascade reaction:

(1) $$\mathrm{Glucose}+O_{2}+H_{2}\overset{\mathrm{GOx}@\mathrm{GA}-\mathrm{Fe}\left(\mathrm{ii}\right)}{\to }\mathrm{Gluconic}\mathrm{acid}\left(H^{+}\right)+H_{2}O_{2}$$

(2) $$H_{2}O_{2}\overset{\mathrm{GOx}@\mathrm{GA}-\mathrm{Fe}\left(\mathrm{ii}\right)}{\to }\cdot \mathrm{OH}$$

(3) $$\mathrm{TMB}\overset{\cdot \mathrm{OH}}{\to }\mathrm{oxTMB}$$

Because bacteria in drinks consume glucose for growth and reproduction, the determination of glucose consumption provides a new method for the detection of bacterial activity. Apple juice was used to explore the utility of this method, assessing both the glucose concentration and bacterial viability. In the detection of actual samples of apple juice, we can detect five common strains with a concentration of 10^7^ cfu, which is an improvement over the previously reported method [27]. Compared with most detection methods, ours involves a simple production method, does not require precision instruments, and has high detection accuracy over wide temperature and pH ranges. Synthesized with common reagents, the nanomaterial GOx@GA-Fe (ii) in this paper has high catalytic activity and stable structure. Otherwise, the low-cost of GOx@GA-Fe (ii) can broaden its scope of application in the detection of food production and processing stages.

## 2. Materials and Methods

### 2.1. Materials and Instrument

Ferrous chloride tetrahydrate (FeCl_2_·4H_2_O), gallic acid (GA), glucose oxidase (GOx), glucose, 3,3′,5,5′-tetramethylbenzidine (TMB), acetic acid (CH_3_COOH), H_3_PO_4_, and NaH_2_PO_4_ were purchased from Sigma-Aldrich; sucrose, fructose, lactose, sodium chloride (NaCl), potassium chloride (KCl), and sodium bicarbonate (NaHCO_3_) were purchased from Aladdin Chemistry Company. Hydrogen peroxidase (H_2_O_2_) was purchased from Alfa Aesar and the apple juice was bought from HuiYuan. The chemicals were all of analytical grade and were used in the original manner without further purification.

Scanning electron microscopy (SEM) was NanoSEM-450 (FEI, Brno, Czech Republic). Transmission electron microscopy (TEM) was JEM-1230 (Japanese Electronics, Tokyo, Japan), which adopts the accelerating voltage of 40–120 kV. The Fourier–transform infrared spectrometer (FITR) was purchased from Germany with the model Vertex70, the visible ultraviolet spectrophotometer were used a UV-2550 spectrophotometer (Shimadzu, Kyoto, Japan). The PB-10 digital pH meter (Sartorius, Goettingen, Germany) was used to test the pH values of the solution.

Strains: *Escherichia coli* (ATCC25922); *Staphylococcus aureus* (ATCC25923); *Salmonella typhimurium* (ATCC50115); *Listeria monocytogenes* (CMCC54004); and *Enterobacter sakazalii* (ATCC29544).

### 2.2. Synthesis of GOx@GA-Fe (ii)

The nanomaterials GOx@GA-Fe (ii) was synthesized by one–pot method; the main process was as follows: 1 mL gallic acid (0.133 mM) and 1 mL FeCl_2_·4H_2_O (0.183 mM) were mixed with continuous shaking at 37 °C for 30 min. The reaction continued at the same condition for 12 h after adding 1 mL glucose oxidase (1.5 mg L^−1^, origin from *Aspergillus niger*). After the mixture was centrifuged at 12,000 rpm for 30 min, the precipitate was collected after being washed twice with 3 mL of deionized water. Scheme 1 demonstrates the synthesis process of GOx@GA-Fe (ii).

### 2.3. GOx@GA-Fe (ii) Activity Assay

Colorimetry is the most common method to determine enzyme activity at present, and TMB method is one of the convenient, fast, and accurate detection methods, so as reaction substrates, glucose and TMB were used to detect the catalytic activity of nanocomposites. First, 20 mM TMB solution was prepared. Then, TMB solution was added (10 μL) to the NaAc-HAc buffer (170 μL, pH = 4.0), followed by a glucose solution of different solubility (15 μL), and finally, GOx@GA-Fe (ii) solution (5 μL) was added to above mix solution. After stirring at 37 °C for 30 min, the absorbance of the mixed solution was measured at 652 nm by UV–Vis spectrophotometer.

### 2.4. Enzymatic Kinetic Analysis 

Kinetic determination is recorded in the case of a different substrate concentration and the corresponding UV spectrophotometer at 652 nm absorbance value. Glucose (determines the material’s affinity for TMB) and TMB (determines the material’s affinity for glucose) solutions in the optimal solubility state are the key to successful kinetic determination. The composites were evaluated by changing the substrate concentration of the phosphate buffer solution (pH = 4.0) at room temperature. Michaelis–Menten constant (K_m_) and the maximum reaction rate (V_max_) were calculated by the double reciprocal method. The kinetic calculation formula is as follows:

(4) $$1/v\;={\;K}_{m}V_{\max}\cdot 1/\left[S\right]\;+\;1/V_{\max}$$

[S] is the substrate (glucose/TMB) concentration and v is the reaction rate. V_max_ is referred to the maximum rate of reaction and can be obtained from the intercept; K_m_ is calculated from the slope.

### 2.5. Detection of Glucose

TMB solution (10 µL, 20 mM) was added to an NaAc buffer (170 µL, pH = 4.0) containing a certain amount of GOx@GA-Fe (ii), and 15 µL of glucose solution (0, 1, 5, 8, 10, 50, 80, 100, 200, 400, 500 µM) was added. The mixed solution was continuously stirred at 37 °C for 20 min, centrifuged at 10,000 rpm for 3 min to remove impurities, and the absorbance was measured under a 652 nm UV–Vis spectrophotometer. GOx is a type of natural enzyme with strong specificity. In this cascade reaction, the glucose oxidase catalyzed the glucose in the first step of the cascade reaction and the product of hydrogen peroxide served as substrate for the subsequent catalytic generation of the hydroxyl radical. Therefore, an interference experiment is vital to assess the specificity of GOx@GA-Fe. Glucose, sucrose, fructose, and lactose are dissolved in ultrapure water, and the final concentration of all of the above sugar solutions was 0.1 mM. Fifteen μL of different sugar solution were added to four enzyme marker holes, then added TMB (10 µL), the NaAc buffer (170 µL) and 5 µL GOx@GA-Fe (ii). The absorbance value at 652 nm was determined after 20 min, 37 °C. The blank group replaced the sugar solution with the buffer, and the other chemical solution and conditions kept the same.

### 2.6. Measurement of Microbial Levels

#### 2.6.1. The Concentration of Sample

Apple juice purchased from the market was diluted with deionized water (water: juice = 9:1) and tested as a sample. Fifteen μL diluted apple juice 2 was added to a 170 μL NaAc-HAc buffer and TMB (10 μL 20 mM), and then 5 μL nanozyme GOx@GA-Fe (ii) was blended with the above solution and reacted for 20 min. After the reaction, the material was centrifuged at 10,000 rpm and the absorbance value of the supernatant was measured at 652 nm. The glucose concentration can be calculated by putting the absorbance value into the standard curve obtained from the above experiments.

#### 2.6.2. The Detection of Bacterial Viability

Escherichia coli (*E. coli*); *Staphylococcus aureus* (*S. aureus*); *Salmonella typhimurium* (*S. typhimurium*); *Listeria monocytogenes* (*L. monocytogenes*); and *Enterobacter sakazalii* (*E. sakazalii*) were selected as detection bacteria. After cultivating these five bacteria in a solid Luria−Bertani (LB) medium 12 h at 37 °C, monocolony inoculated in the LB medium (40 mL) at 37 °C for 12 h at 180 rpm. The bacterial suspension was washed by PBS three times. The bacterial suspensions were finally diluted to 10^7^ for detection.

Each prepared 10^7^ bacterial suspension was divided into two parts. One part was placed in a 4 degree refrigerator for preservation, and the other part was put in a high-pressure sterilization pot at 121 °C for inactivation. The apple juice purchased in the market was diluted with deionized water, and the volume ratio of apple juice to deionized water was 1:9. The diluted apple juice was mixed with bacteria in a mixture ratio of 2:1, incubated 12 h, and then the mixture was subsequently removed by centrifuged (10,000 rpm, 5 min). The supernatant was added to the GOx@GA-Fe (5 µL), TMB (10µL), and an NaAc-HAc buffer solution (170 µL). After 20 min of reaction, the absorbance intensity was tested by a UV-visible instrument. 

## 3. Results and Discussion

### 3.1. Structural and Morphological Studies of Nanostructured Materials

**Figure 1:**
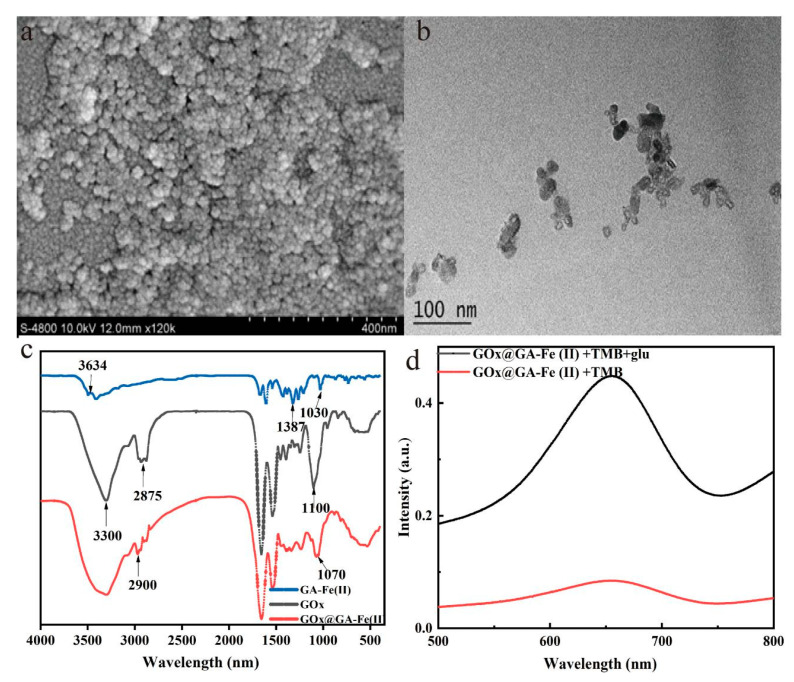
(**a**)The SEM image of GOx@GA-Fe (ii); (**b**) the transmission electron microscopy image of GOx@GA-Fe (ii); (**c**) the Fourier Transform infrared spectroscopy images of GOx@GA-Fe (ii), GOx, GA-Fe (ii); and (**d**) the characteristic reaction of GOx@GA-Fe (ii) to the substrate glucose.

Scanning and transmission electron microscopy were used to examine the structure of the nanocomposite. A scanning electron microscopy image of the sample shows spherical 20 nm particles with a uniform distribution and size (Figure 1).Transmission electron microscopy images of the composite material in Figure 1 and Figure S1 show that the GOx@GA-Fe (ii) nanoparticles are ellipsoidal. Through these images, it is shown that GOx@GA-Fe (ii) is mainly composed of two parts (light and dark region) [28,29]. The structures of the prepared aqueous solutions GA-Fe (ii), GOx, and GOx@GA-Fe (ii) were characterized by FTIR spectroscopy, as shown in Figure 1, and the spectral bands at 4000–400 cm^−1^. The figure of GA-Fe includes obvious peaks, such as 3634 cm^−1^, 1387 cm^−1^, and 1250 cm^−1^. The absorption peak at 3634 cm^−1^ comes from the O-H of GA, and 1387 cm^−1^ is the stretching vibration of the corresponding C-O bond. GOx has strong absorption peaks at 1100 cm^−1^ and 3300 cm^−1^, which is the stretching vibration of C-C and O-H bonds [17]. Though the peak at 2875 cm^−1^ is not a strong peak of GOx@GA-Fe (ii), it is a characteristic peak different from GA-Fe. After the GOx and GA-Fe compound to form GOx@GA-Fe (ii), containing all the characteristic peaks of GA and GOx, which shows peak attenuation or deviation, such as peak attenuation of 1100 cm^−1^ and 2875 cm^−1^. In addition, 2900 cm^−1^ and 1070 cm^−1^ is an obvious offset peak, which reflects the fracture and recombination of bonds in the recombination process. The analysis results of the FTIR spectroscopy showed that all the characteristic peaks of GOx appeared in synthesized nanomaterial GOx@GA-Fe (ii), which indicates the undamaged structure of glucose oxidase during synthesis. The material also showed a unique Fe-MOF structure peak. In summary, the preparation of the nanomaterial GOx@GA-Fe (ii) was relatively successful, and glucose oxidase was successfully combined with GA-Fe structure by the one–pot method. 

### 3.2. Optimization of Conditions

For every enzyme (both natural and artificial), viability measurement is very important and it is vital to assess the catalytic capacity of the cascade enzyme. The nanozyme GOx@GA-Fe (ii) is supported by the above characterization methods. To further verify the feasibility of the cascade method, the ability of GOx@GA-Fe (ii) to catalyze the generation of hydrogen peroxide (H_2_O_2_) from glucose and the oxidization of TMB (blue) was investigated. As shown in Figure 1, in the absence of glucose, the absorbance at 652 nm is very low, which instructs the TMB to not oxidize, which further showed that no H_2_O_2_ was generated, and as it turns out, there is no substrate of glucose oxidase from the other side. On the contrary, in the presence of glucose and TMB, a significant increase in absorbance was observed at 652 nm with the help of UV-visible, revealing that GOx@GA-Fe (ii) could oxidase glucose to produce H_2_O_2_, which can be transformed to an hydroxyl radical under GA-Fe (ii), and finally oxidase TMB to oxTMB (blue). Figure S2 in Supplementary Material can more intuitively show the difference between the presence and absence of glucose. We can directly see the difference with naked eyes. One is transparent (without glucose), whereas the other is dark blue (with glucose). The results show that this method can be used to detect glucose. 

Nanozymes will have better catalytic activity under suitable conditions. Therefore, we tested the GOx@GA-Fe (ii) under varying temperatures, pH levels, and times. We set 7 points between 30 and 60 degrees Celsius at 30, 35, 40, 45, 50, 55, 60 °C, respectively. The temperature gradient showed the best enzyme activity at 35–40 °C (Figure S3). The optimum yield of glucose oxidase origin from *A. niger.* is about 27–37 °C [30]. In order to maximize the role of GOx, we set 37 °C as the catalytic temperature in this paper. Trough analysis we can conclude that the optimum temperature changes from 27–37 °C to 35–40 °C, GA-Fe (ii) play an important role, we speculated that GA-Fe (ii) not only protects the sensitive enzyme from high/low temperature damage, but also changes the best reaction temperature. In addition, we varied the pH from 1 to 9 and found that enzyme activity was greatest at pH 4 (Figure S3), which is entirely different from the common optimal pH between 6 and 7. According to relevant literature reports, when pH is not in the optimal range, the viability of GOx declines dramatically [31], so the explanation for the wider pH range after composite process is that the GOx is buried in the structural gap of Ga-Fe (ii), and the narrow space prevents the secondary structure of GOx from stretching and deforming, which resulted in the loss of catalytic activity. Then, we varied the reaction time from 0 to 60 min and found that the enzyme activity increased rapidly up to 30 min, after which it leveled off (Figure S3). Because the reaction process exceeded 80% within 20min, 20 min was adopted as the end point of the reaction in subsequent tests. Taking various factors into consideration, the best conditions for nanomaterial GOx@GA-Fe (ii) to play a catalytic role as sensor probes are as follows: 37 °C, pH = 4, and 20 min.

The stability of the nanozyme was also tested by keeping it in a refrigerator at 4 °C and regularly removing and testing aliquots. The material retained approximately 70% of its initial activity at 60 days after synthesis (Figure S4). Glucose oxidase is usually stored in −20 degrees to keep its original catalysis capacity, which has higher requirements for transportation and storage. Based on the above temperature and pH endurance capacity of the composite nanomaterial GOx@GA-Fe (ii), it is obvious that GA-Fe (ii) forms an excellent protective structure for vulnerable GOx. This brings great convenience for the storage, transportation, and use of the nanozyme GOx@GA-Fe (ii).

### 3.3. Investigation of Catalytic Activity of the Nanozyme GOx@GA-Fe (ii)

**Figure 2:**
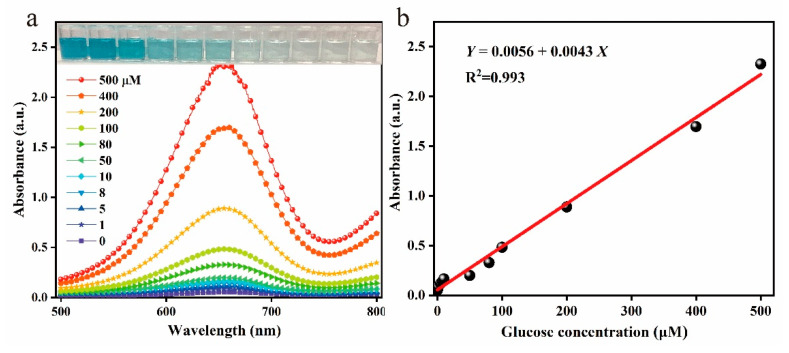
The absorbance value of oxTMB after glucose was catalyzed by (**a**) GOx@GA-Fe (ii) and (**b**) its linear regression analysis.

To determine the continuous catalytic activity of the nanocomposite enzyme GOx@GA-Fe (ii), glucose was used as the substrate under the above optimal conditions. The enzyme first oxidizes glucose to gluconic acid and H_2_O_2_ and then catalyzes H_2_O_2_ to yield ·OH, which then reacts with TMB to form blue oxidized TMB. We evaluated the suitability of the material as a glucose sensor platform. With increasing glucose concentrations, the ultraviolet (UV) absorption intensity also gradually increased. UV spectrophotometry was used to measure the UV–Vis absorption of liquid containing 1–500.0 µM glucose (Figure 2). The linear relationship between the absorbance (*Y*) and glucose concentration (*X*) of the solution had a correlation coefficient of R^2^ = 0.993.

The detection limit was 0.43 µM, and the signal-to-noise ratio was 3 (Figure 2). The quantitative determination of the glucose content was realized. Based on the glucose standard curve and formula obtained from the above experiments, we tried to explore the glucose concentration in actual samples further. All testing steps were consistent with the standard glucose solution except that the glucose solution was replaced with the sample solution. The detection capability of the nanomaterial in this paper is superior to most existing probes in the market when the detection limit and detection range were considered comprehensively. In order to explain this point more clearly, we collected some recent detection capabilities of synthetic materials and summarized them into a table (Table S1). It can be seen from the table that our material detection level is relatively high, which provides a new high-precision detection probe for the field of rapid detection.

### 3.4. The Kinetic Curve and Selectivity of GOx@GA-Fe (ii)

**Figure 3:**
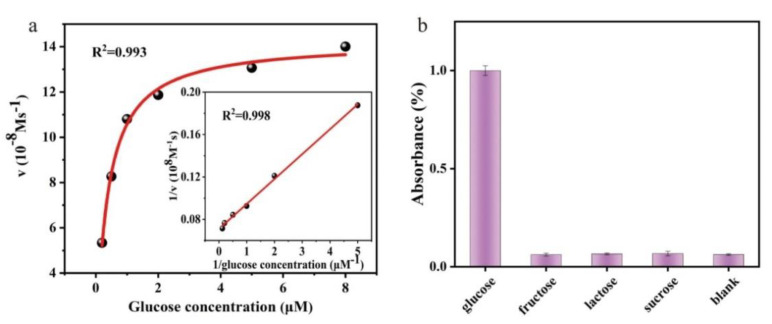
Response range of catalytic glucose with GOx@GA−Fe (ii) (**a**) Selectivity for glucose detection by monitoring the relative activity and (**b**). The error bars represent the standard deviation of three measurements.

To examine the catalytic performance of GOx@GA-Fe (ii), the steady-state kinetics of the TMB system were measured according to the Michaelis–Menten curve. The maximum initial velocity (V_max_) and Michaelis constant (K_m_) were calculated using the Lineweaver–Burk equation (Figure 3). As expected, GOx@GA-Fe (ii) showed good affinity for glucose and significant catalytic activity toward the substrate, indicating that GA-Fe is a good peroxidase. Compared with free GOx, the increased Km of GOx@GA-Fe (ii) indicates that the enzyme has better substrate affinity and mass transfer ability. When glucose and TMB were used as substrates, K_m_ values were 3.456 mM and 2.334 mM [32], respectively, which were lower than the natural enzyme GOx [33]. The results indicated that the nanozyme GOx@GA-Fe (ii) has a high affinity toward TMB and glucose. On the other hand, the calculated V_max_ values are 14.234 × 10^−8^ M s^−1^ for glucose and 6.161 × 10^−8^ M s^−1^ for TMB. The results clearly showed that the GOx@GA-Fe (ii) prepared by a one–pot method equipped with good cascade enzyme-like activity.

To verify the reliability of our glucose colorimetric detection method, we also selected four common sugars, excluding glucose, for determining whether nanomaterials GOx@GA-Fe (ii) are specific. The final concentration of each sugar solution is 0.1 mM, together with a blank negative control (deionized water). As shown in Figure 3, the GOx@GA-Fe (ii) reacted only at the presence of glucose, and without obvious phenomenon with the other four kinds of sugar, implying that those sugars would have no effect on the selective determination of glucose. This result is in accordance with expectations, for the first step of this cascade catalysis is about oxidation reaction dominated by glucose oxidase, which is entirely absorbed in glucose. GOx was not reacting with other sugar, excluding glucose, which determines that the cascade probe GOx@GA−Fe (ii) can only act on function in the presence of glucose. It is precisely because of this that the phenomenon could be used in various fields, particularly in food detection. As well-known food systems can be so very intricate that many conventional detection methods cannot effectively reflect real food situations, other ingredients in the food can interfere with detection. The specific system is very important when GOx@GA−Fe (ii) was used as a probe in a food detection process.

### 3.5. Real Sample Detection

To further measure the practicality of nanozyme GOx@GA−Fe (ii) based on colorimetric method, the beverage industry, which produces many drinks containing a variety of nutrients, including vitamins and essential trace elements. With the popularity of these drinks comes the risk of obesity and diabetes due to their sugar content. Therefore, to improve the accuracy and precision of the determination of the sugar content of drinks, we examined samples of apple juice.

**Figure 4:**
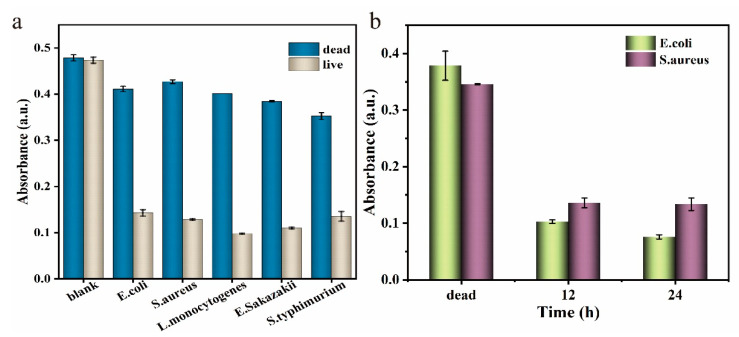
(**a**) The absorbance value of different strains at 652 nm to observe the bacterial viability. (**b**) Detection of bacterial activity (*E. coli* and *S. aureus*) after different incubation time.

As shown in Figure 4, the absorbance of the diluted juice was approximately 0.48. Substituting this value into the above formula, the glucose concentration of the diluted apple juice was calculated as 110.33 µM; thus, the original glucose content was approximately 1.9872 mg/100 mL. This is in the glucose concentration range of the apple juice [34]. This proves that our method is reasonable to detect the concentration of apple juice. In order to reduce the measurement error caused by raw materials, especially the raw liquid of apple juice having a pale yellow color, and because this method is based on color reaction to detect glucose content, it is necessary to reduce the interference caused by the color of the actual sample on the reaction. Common soft drinks have color due to the food coloring added or brought by the raw material, and dilution can effectively reduce the concentration of the material. After measuring the concentration of the diluted material multiplied by the dilution factor the glucose content of the raw material. Actual sample pretreatment was conducted as far as possible to ensure reliability of results.

**Scheme 2:**
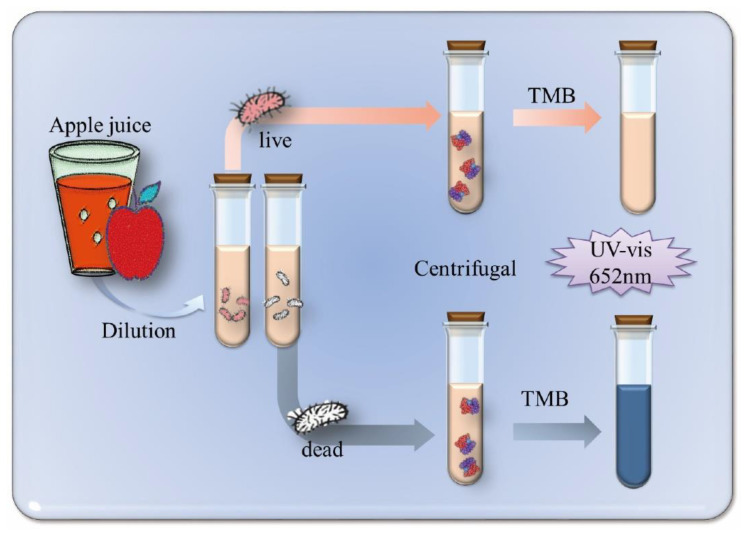
The scheme to illustrate the colorimetric detection process of microbial activity.

Herein, we provide a method to detect the activity of bacteria in actual samples for broadening the nanozyme GOx@GA-Fe (ii) in practical application. Apple juice contains a large amount of glucose and foodborne microorganisms may use these glucose as its energy metabolism and breeding material through the comparison on the samples before and after the sterilization intensity of light absorption in the UV–Vis to determine whether the glucose in the apple juice had been consumed by a foodborne microbial. The small difference illustrates that the juice was not contaminated, simultaneously if the absorbance value of prior sterilization is low, which indicates the probability of the apple juice being polluted is higher. Comparing the absorbance of tubes containing live and dead bacteria, our method was useful for detecting foodborne pathogens (Scheme 2). After incubating diluted apple juice with four groups of bacterial suspension and the control group for 12 h, the bacteria were removed by centrifugation, and the glucose content of supernatant was detected. The results were the same among tubes containing *Escherichia coli*, *Staphylococcus aureus*, *Listeria* sp., and *Salmonella typhimurium*, including both Gram-positive and Gram-negative species and the blank control (Figure 4). The presence of living bacteria decreased the absorbance, whereas dead bacteria had no obvious effect (Figure 4). The material can indeed detect bacteria in actual samples through glucose consumption. Compared with previous detection methods, such as the proposal of Ding et al. [26], our method reduces the concentration of detected bacteria by an order of magnitude without increasing the detection time. In order to further explore the detection time of the material, we took *Escherichia coli *and *Staphylococcus aureus* as examples with the results shown in Figure 4. After coculture, centrifugation was carried out and the supernatant was taken for measurement. It was found that after 12 h, the absorbance decreased significantly, indicating that the microorganisms had utilized glucose. At the same time, we found no significant difference between 24 h and 12 h; glucose having been used up or the microorganism entering the decay stage and reducing the glucose consumption are probably the main reasons. The glucose content of the control group was lower than that of the original apple juice (absorption value was about 4, lower than 4.8). We speculated that the reasons for this situation were as follows: the bacteria were not completely killed or the reaction system was contaminated by bacteria in the environment during incubation and reaction. There was no obvious difference between Gram-positive and -negative bacteria. This is the advantage of the GOx@GA-Fe (ii); the nanozyme can detect a variety of foodborne bacteria. In the daily food quality inspection process, because the food contains a lot of nutrients, it is very suitable for the survival and reproduction of microbial mold. Rapid and efficient detection is very important, and determining the presence of bacteria is an essential part of the detection. One–pot synthesis nanozyme GOx@GA-Fe (ii) proved to be a good detection material.

## 4. Conclusions

In summary, we prepared a cascade composite nanozyme GOx@GA-Fe (ii) by combined glucose oxidase and peroxidase activities. The stability of GOx increased through embedding in the surface of GA-Fe (ii). The cascade nanozyme with good specialized sensibility is used as a colorimetric probe to assess the glucose content in complex food systems. In addition, combined with glucose consumption by microbial metabolism, the sensor was successfully used for microbial detection. Under the appropriate conditions (37 °C, pH = 4), the enzyme first converts glucose to gluconic acid and H_2_O_2_ and then catalyzes the generation of ·OH. The hydroxyl radicals oxidize TMB, the absorbance of which can be measured by UV–Vis spectrophotometry to determine the glucose content. We applied this colorimetric method to detect the microbial viability of five common foodborne bacterial in apple juice samples. The glucose content and microbial activity of foods can be assessed simultaneously, providing a new method for assessing food quality.

## Data Availability

Not applicable.

## Supplementary Materials

The following are available online at https://www.mdpi.com/article/10.3390/foods10112596/s1, Figure S1: The morphology of nanomaterials with different sizes; Figure S2: Optimization of material reaction conditions; Figure S3: The steady-state kinetics of nanozyme; Figure S4: GOx@GA-Fe (ii) stability detection time span of 1 to 60 days; Figure S5: The steady-state kinetics of GOx@GA-Fe (II)/glucose (a) and GOx@GA-Fe (II)/TMB (b). Table S1: Comparison of the ability of different nanometer synthases to detect glucose.
