# Supplementary material for: Natural Products Self-Assembled Nanozyme for Cascade Detection of Glucose and Bacterial Viability in Food
Source: Foods. 2021 Oct 27;10(11):2596. doi: 10.3390/foods10112596 (PMC8618841; doi:10.3390/foods10112596)
Supplement: Supplementary file 1 [file foods-10-02596-s001.zip › foods-1405420-supplementary.pdf]

Supplementary Materials: Natural Products Self-Assembled Nanozyme for Cascade Detection of Glucose and Bacterial Viability in Food

Qiuping Zhang, Yi Kang, Hao Sun, Yanmin Liang, Zehui Su, Jie Dan, Linpin Luo, Tianli Yue,

Jianlong Wang *, Wentao Zhang *

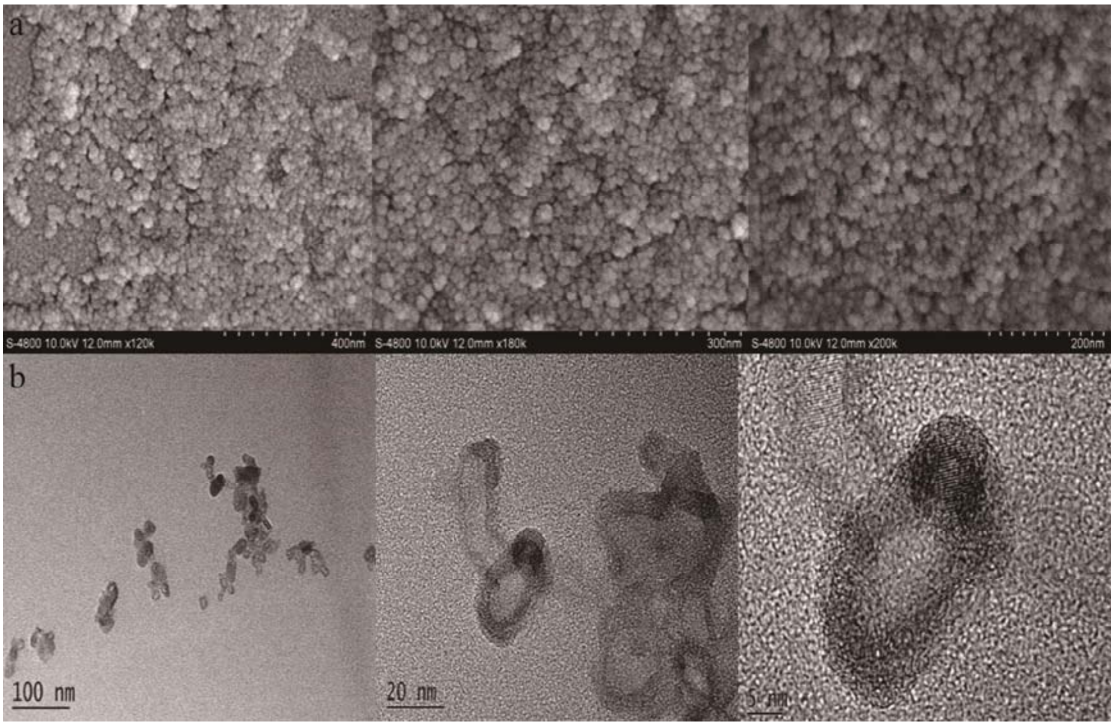

Figure S1. The morphology of nanomaterials with different sizes, including SEM image (a); and TEM image (b).

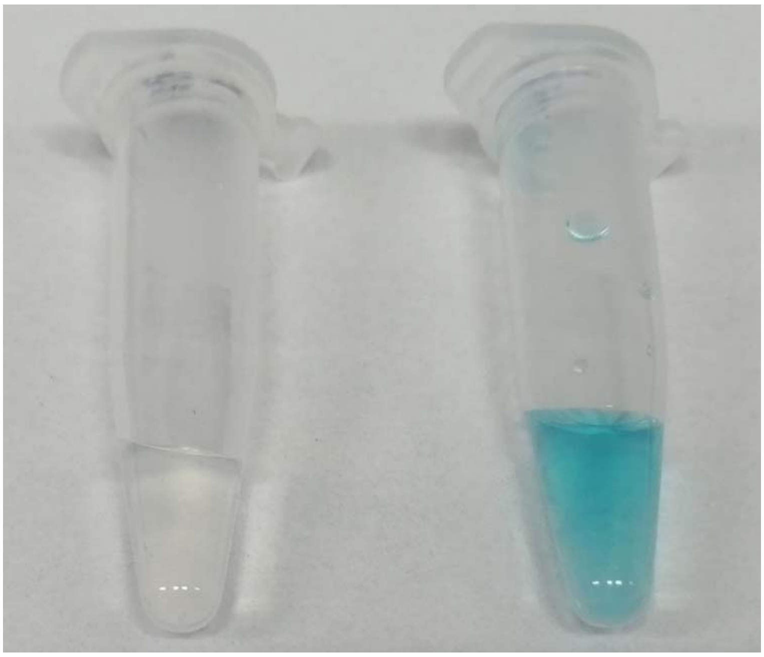

Figure S2. Effect of the presence of glucose on the chromogenic reaction: GOx@GA-Fe (II) +TMB (a); GOx@GA-Fe (II) +TMB +Glucose (b). The concentration of Glucose was 100 μL, and the other conditions were the same.

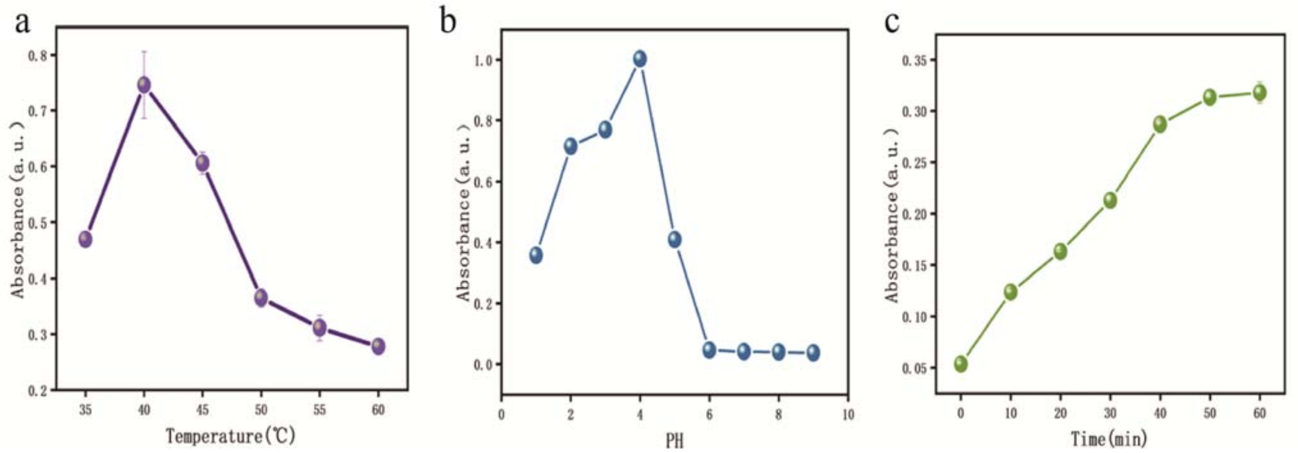

Figure S3. Optimization of material reaction conditions, including temperature (a); pH(b) and reaction time(c).

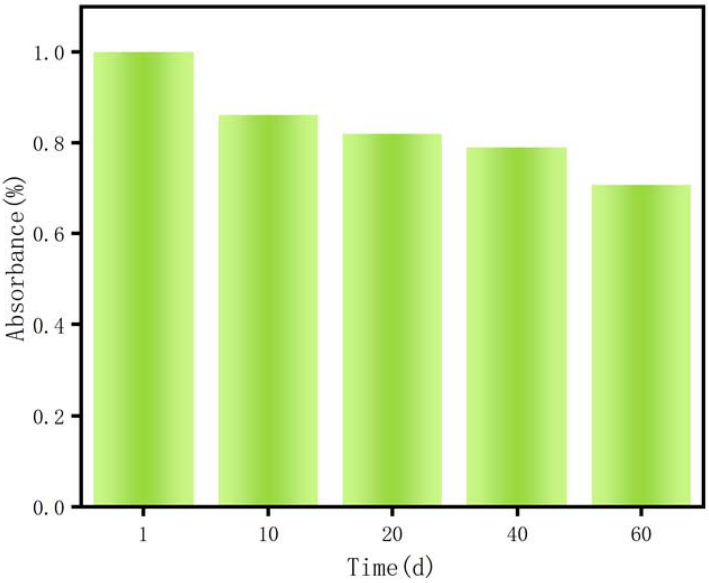

Figure S4. Stability detection of GOx@GA-Fe (ii) from1 to 60 days.

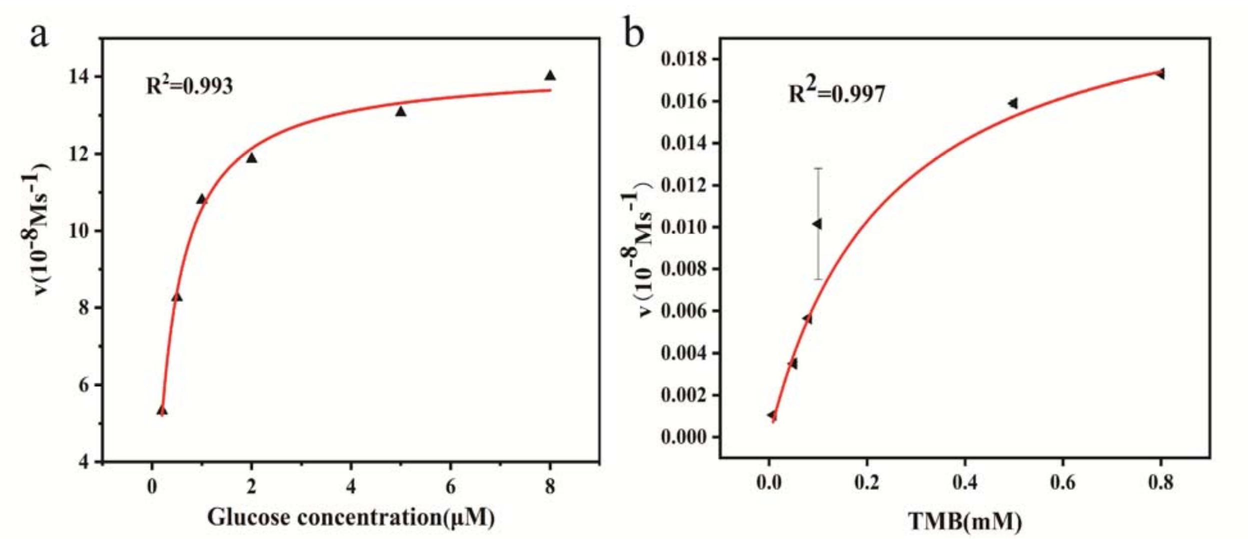

Figure S5. The steady-state kinetics of GOx@GA-Fe (II)/glucose(a) and GOx@GA-Fe (II) /TMB(b).

Table S1 Comparison of the ability of different nanometer synthases to detect glucose.

Nanozyme	Linear range(μM)	LOD(μM)	Ref
**GOx@CuBDC**	10−500	4.1	[1]
**GOx@ZIF-8(NiPd)**	10−300	9.2	[2]
**GOx@MOF-545(Fe)**	0.5−100	0.28	[3]
**H_2TCPP-NiO + GOx**	50−500	20	[4]
**2D Au NPs/Cu-TCPP(Fe**	10−300	8.4	[5]
**Au@Ag + GOx**	50−20,000	39	[6]
**SiO2/Imi/Pt/GOx**	100−800	163	[7]
**Chitosan-Au**	1-5000	3	[8]
**CNT-CS-Au**	6–5000	3	[9]
**Au@Ag NRs**	50–20,000	39	[6]
**Fe3O4-Au-CS**	3–570	1.2	[10]
**Porphyrin-ZnS**	50–500	36	[11]
**Fe3O4@CN-GQDs**	6–100	2	[12]
**Humic acid-Cu**	1–100	0.68	[13]
**Carbon nanodots**	1–50	0.4	[14]
**AuNPs@C.CNF**	1–60	0.67	[15]
**GOx@GA-Fe(II)**	0.1-500	0.43	This paper

Reference

1 Cheng, X.; Zheng, Z.; Zhou, X.; Kuang, Q., Metal–Organic Framework as a Compartmentalized Integrated Nanozyme Reactor to Enable High-Performance Cascade Reactions for Glucose Detection. ACS Sustainable Chemistry & Engineering 2020, 8 (48), 17783-17790.

2 Wang, Q.; Zhang, X.; Huang, L.; Zhang, Z.; Dong, S., GOx@ ZIF‐8 (NiPd) nanoflower: An artificial enzyme system for tandem catalysis. Angewandte Chemie 2017, 129 (50), 16298-16301.

3 Zhong, X.; Xia, H.; Huang, W.; Li, Z.; Jiang, Y., Biomimetic metal-organic frameworks mediated hybrid multi-enzyme mimic for tandem catalysis. Chemical Engineering Journal 2020, 381, 122758.

4 Liu, Q.; Yang, Y.; Li, H.; Zhu, R.; Shao, Q.; Yang, S.; Xu, J., NiO nanoparticles modified with 5, 10, 15, 20-tetrakis (4-carboxyl pheyl)-porphyrin: promising peroxidase mimetics for H_2O_2 and glucose detection. Biosensors and Bioelectronics 2015, 64, 147-153.

5 Huang, Y.; Zhao, M.; Han, S.; Lai, Z.; Yang, J.; Tan, C.; Ma, Q.; Lu, Q.; Chen, J.; Zhang, X., Growth of au nanoparticles on 2D metalloporphyrinic metal‐organic framework nanosheets used as biomimetic catalysts for cascade reactions. Advanced Materials 2017, 29 (32), 1700102.

6 Han, L.; Li, C.; Zhang, T.; Lang, Q.; Liu, A., Au@ Ag heterogeneous nanorods as nanozyme interfaces with peroxidase-like activity and their application for one-pot analysis of glucose at nearly neutral pH. ACS applied materials & interfaces 2015, 7 (26), 14463-14470.

7 Du, Y.; Gao, J.; Liu, H.; Zhou, L.; Ma, L.; He, Y.; Huang, Z.; Jiang, Y., Enzyme@

silica nanoflower@ metal-organic framework hybrids: A novel type of integrated nanobiocatalysts with improved stability. Nano Research 2018, 11 (8), 4380-4389.

8 Jiang, C.; Zhu, J.; Li, Z.; Luo, J.; Wang, J.; Sun, Y., Chitosan–gold nanoparticles as peroxidase mimic and their application in glucose detection in serum. RSC advances 2017, 7 (70), 44463-44469.

9 Wang, F.; Ding, X.; Niu, X.; Liu, X.; Wang, W.; Zhang, J., Green preparation of core-shell Cu@ Pd nanoparticles with chitosan for glucose detection. Carbohydrate Polymers 2020, 247, 116647.

10 Li, J.; Yuan, R.; Chai, Y., Simple construction of an enzymatic glucose biosensor based on a nanocomposite film prepared in one step from iron oxide, gold nanoparticles, and chitosan. Microchimica Acta 2011, 173 (3-4), 369-374.

11 Liu, Q.; Chen, P.; Xu, Z.; Chen, M.; Ding, Y.; Yue, K.; Xu, J., A facile strategy to prepare porphyrin functionalized ZnS nanoparticles and their peroxidase-like catalytic activity for colorimetric sensor of hydrogen peroxide and glucose. Sensors and Actuators B: Chemical 2017, 251, 339-348.

12 Li, Q.; Tang, G.; Xiong, X.; Cao, Y.; Chen, L.; Xu, F.; Tan, H., Carbon coated magnetite nanoparticles with improved water-dispersion and peroxidase-like activity for colorimetric sensing of glucose. Sensors and Actuators B: Chemical 2015, 215, 86-92.

13 Wang, N.; Li, B.; Qiao, F.; Sun, J.; Fan, H.; Ai, S., Humic acid-assisted synthesis of stable copper nanoparticles as a peroxidase mimetic and their application in glucose detection. Journal of materials chemistry B 2015, 3 (39), 7718-7723.

14 Shi, W.; Wang, Q.; Long, Y.; Cheng, Z.; Chen, S.; Zheng, H.; Huang, Y., Carbon nanodots as peroxidase mimetics and their applications to glucose detection. Chemical Communications 2011, 47 (23), 6695-6697.

15 Alle, M.; Park, S. C.; Bandi, R.; Lee, S.-H.; Kim, J.-C., Rapid in-situ growth of gold nanoparticles on cationic cellulose nanofibrils: Recyclable nanozyme for the colorimetric glucose detection. Carbohydrate Polymers 2021, 253, 117239.
